# A Comparative Analysis on Impact of Extraction Methods on Carotenoids Composition, Antioxidants, Antidiabetes, and Antiobesity Properties in Seagrass *Enhalus acoroides*: In Silico and In Vitro Study

**DOI:** 10.3390/md22080365

**Published:** 2024-08-12

**Authors:** Raymond Rubianto Tjandrawinata, Fahrul Nurkolis

**Affiliations:** 1 Center for Pharmaceutical and Nutraceutical Research and Policy, Atma Jaya Catholic University of Indonesia, Jakarta 12930, Indonesia; 2 Department of Chemistry, Faculty of Mathematics and Natural Science, Universitas Padjadjaran, Sumedang 45363, Indonesia; fahrul.nurkolis.mail@gmail.com

**Keywords:** ultrasound-assisted extraction, microwave-assisted extraction, seagrass, marine product, carotenoids, antioxidants, antidiabetes, antiobesity, green extraction

## Abstract

*Enhalus acoroides*, a tropical seagrass, is known for its significant contribution to marine ecosystems and its potential health benefits due to bioactive compounds. This study aims to compare the carotenoid levels in *E. acoroides* using green extraction via ultrasound-assisted extraction (UAE) and microwave-assisted extraction (MAE) and to evaluate the biological properties of these extracts against oxidative stress, diabetes, and obesity through in silico and in vitro analyses. *E. acoroides* samples were collected from Manado City, Indonesia, and subjected to UAE and MAE. The extracts were analyzed using UHPLC-ESI-MS/MS to identify carotenoids, including β-carotene, lutein, lycopene, β-cryptoxanthin, and zeaxanthin. In silico analysis was conducted to predict the compounds’ bioactivity, toxicity, and drug-likeness using WAY2DRUG PASS and molecular docking with CB-Dock2. The compounds C3, C4, and C7 demonstrated notable interactions, with key metabolic proteins and microRNAs, further validating their potential therapeutic benefits. In vitro assays evaluated antioxidant activities using DPPH and FRAP assays, antidiabetic properties through α-glucosidase and α-amylase inhibition, and antiobesity effects via lipase inhibition and MTT assay with 3T3-L1 cells. Results indicated that both UAE and MAE extracts exhibited significant antioxidant, antidiabetic, and antiobesity activities. MAE extracts showed higher carotenoid content and greater biological activity compared to UAE extracts. These findings suggest that *E. acoroides*, mainly when extracted using MAE, has promising potential as a source of natural bioactive compounds for developing marine-based antioxidant, antidiabetic, and antiobesity agents. This study supplements existing literature by providing insights into the efficient extraction methods and the therapeutic potential of *E. acoroides* carotenoids.

## 1. Introduction

Seagrasses, such as *Enhalus acoroides*, are essential components of marine ecosystems, providing various ecological services and serving as a source of bioactive compounds with potential health benefits [1]. This species is a significant component of tropical seagrass beds, characterized by its large size and common occurrence, contributing substantially to the total seagrass biomass [2]. It is known for its coarse, robust, and deep-penetrating rhizomes, which distinguish it from other seagrass species [3]. *E. acoroides* has been extensively studied for its phytochemical composition, antioxidant properties, and potential applications in combating diseases such as diabetes and obesity [4,5]. Moreover, *E. acoroides* has shown potential in inhibiting α-glucosidase, which can be beneficial in diabetes management [6]. The seagrass has also exhibited antioxidant activities, hindering the formation and promoting the dispersion of medical biofilms [7]. Additionally, *E. acoroides* has been investigated for its chemo-preventive and therapeutic efficacy against hepatocellular carcinoma [8].

The impact of extraction methods on seagrass composition and properties is crucial. For instance, a study investigated the restoration performance of *E. acoroides* using different methods, emphasizing the importance of selecting appropriate techniques for seagrass transplantation [9]. The extraction methods used to obtain bioactive compounds from *E. acoroides* involve techniques such as maceration with different solvents such as methanol, ethyl acetate, and n-hexane [10]. These methods are essential for isolating and concentrating the beneficial compounds present in the seagrass. Green extraction techniques, such as ultrasound-assisted extraction (UAE) and microwave-assisted extraction (MAE), have gained significant attention in recent years due to their environmentally friendly nature and ability to produce high-quality extracts using less solvent, time, and energy [11]. These methods have been utilized to convert organic residues into valuable products with enhanced properties, such as functional foods, cosmetics, and bioactive compounds [12]. In the context of polyphenol extraction, modern green extraction techniques such as UAE and MAE have been highlighted as alternatives to conventional methods, offering improved efficiency and reduced extraction time and solvent consumption [13]. MAE, in particular, has been identified as a promising green extraction method due to its ability to provide rapid energy transfer and simultaneous heating of biological materials and solvents, resulting in higher extraction yields and antioxidant activity compared to other methods, such as maceration [14].

Physically, *E. acoroides* is green seagrass, which suggests the presence of chlorophyll or xanthophyll pigments associated with carotenoids. However, no research has successfully reported the carotenoid levels in *E. acoroides* or explored its biological activities, such as antioxidant, antidiabetic, and antiobesity properties. The current study aims to compare the carotenoid levels in *E. acoroides* using two different extraction methods, UAE and MAE. Additionally, it will evaluate the biological properties of these extracts in combating oxidative stress, diabetes, and obesity through in silico and in vitro analyses. This study aims to supplement the literature on new sources of carotenoids and provide insights into the development of new marine-based antioxidant, antidiabetic, and antiobesity agents, which have never been reported previously.

## 2. Results

### 2.1. Quantitation and Identification of E. acoroides Carotenoids Content

Utilizing an Orbitrap analyzer, mass spectrometry (MS) was conducted to determine the presence of carotenoids in *E. acoroides*. The identities of these compounds were verified through comparison with conventional standards (see Table 1). The xanthophylls successfully identified in *E. acoroides* included fucoxanthin, lutein, astaxanthin, canthaxanthin, zeaxanthin, and β-cryptoxanthin. The MS system, operated in ESI negative-positive mode, effectively ionized xanthophylls but failed to ionize carotene hydrocarbons, such as β-carotene and lycopene. As a result, the presence of β-carotene in *E. acoroides* was detected and quantified using ultra-high-performance liquid chromatography (UHPLC) with a diode array detector. Consequently, β-carotene, listed as the sixth carotenoid in Table 1, was successfully identified and quantified.

Further quantification of carotenoid levels in *E. acoroides* is provided in Table 2. Two green extraction methods were used to extract carotenoids from *E. acoroides*: *E. acoroides*—ultrasound-assisted extraction (E-UAE) and *E. acoroides*–microwave-assisted extraction (E-MAE), both utilizing water as the solvent. It should be noted that each carotenoid may have a different solubility profile, and both of these green extraction methods extract carotenoids at different rates. Fucoxanthin, lutein, and β-carotene are the three most dominant carotenoids in E-UAE, with 7.65, 9.73, and 8.58 mg/100 g concentrations, respectively. In total, the six carotenoids in E-MAE are present at significantly lower levels compared to the E-UAE extract (*p* < 0.05; Table 2). Interestingly, the carotenoid levels of canthaxanthin in E-UAE and E-MAE do not differ significantly (3.51 and 2.04 mg/100 g, respectively) (*p* > 0.05). Therefore, both E-UAE and E-MAE green extraction methods yield six carotenoids at different levels in *E. acoroides.* To obtain abundant amounts of carotenoids, the ultrasound-assisted extraction (E-UAE) method is recommended. Fucoxanthin, lutein, and β-carotene are the three most dominant carotenoids found in *E. acoroides.*

### 2.2. In Silico Analysis

#### 2.2.1. Predicting the Activities of Bioactive Compounds, Analyzing Toxicity, and Assessing Drug-Likeness

In the in silico analysis, probability activity (Pa) screening was performed based on structure–activity relationship (SAR) predictions. It was found that the seven carotenoids observed in *E. acoroides* have potential as lipid metabolism regulators and antiobesity agents, with Pa values greater than 0.4 (data presented in Table 3). Among these seven carotenoids, only three (C3, C4, and C7) had a predicted LD50 value greater than 3, making them suitable candidates for advancing to the stage of molecular docking simulation, despite all of them being classified as having rejection classes based on ADMET analysis.

#### 2.2.2. Molecular Docking Simulations

The results of the molecular docking simulations targeting specific genes or proteins are presented in Table 4. This table illustrates the efficacy of the identified compounds C3, C4, and C7 in their molecular docking interactions with several significant proteins: iNOS (3E7G), lipase (1LPB), α-glucosidase (3L4Y), and α-amylase (2QV4). Acarbose was used as a control substance to evaluate the antidiabetic activity, specifically targeting α-glucosidase and α-amylase. Orlistat served as a control to assess the antiobesity effects specifically targeting lipase. Lastly, S-ibuprofen was used as a control to assess the inflammation and antioxidant activity specifically targeting iNOS. The affinity values are also presented in Table 4. The results demonstrate that the compounds C3, C4, and C7 consistently display higher binding affinity values than the established threshold values for controls across all the proteins.

The assessment was conducted by evaluating the substances’ ability to impede signal binding to specific proteins. Table 5 provides a visual depiction of the interaction between amino acids and the compounds identified from other extracts. Compounds other than C3 and C4, as well as the controls targeting specific receptor proteins, are listed in Table S1.

### 2.3. In Vitro Analysis

#### 2.3.1. Antioxidants: FRAP and DPPH with Trolox Control

**Figure 1:**
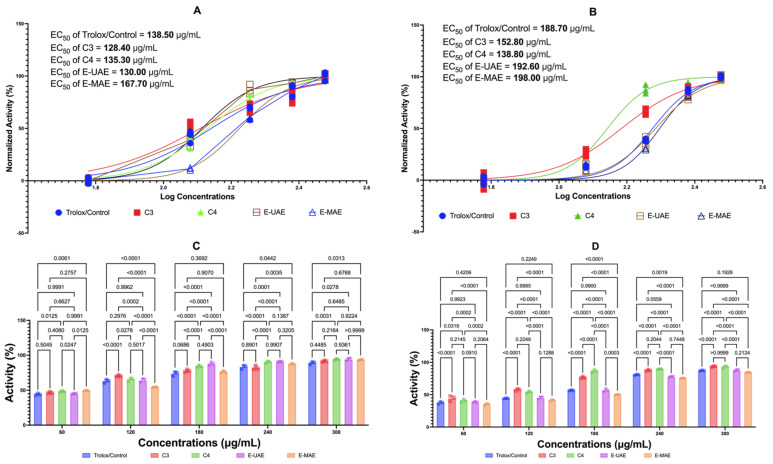
Antioxidant activity of *E. acoroides*. (**A**) EC_50_ of FRAP. (**C**) The difference in antioxidant activity in the FRAP test based on two-way ANOVA. (**B**) EC_50_ of DPPH inhibition activity. (**D**) Different antioxidant activity in the DPPH test based on two-way ANOVA. C3, astaxanthin; C4, canthaxanthin; E-UAE: *E. acoroides*—ultrasound-assisted extraction; E-MAE: *E. acoroides*—microwave-assisted extraction.

Figure 1 displays the antioxidant effects of C3, C4, E-UAE, and E-MAE. In the FRAP test, across all concentrations, C3 tends to have similar antioxidant activity to Trolox while C4, E-UAE, and E-MAE have better antioxidant activity than Trolox. While in the DPPH assay, only C3 and C4 display stronger antioxidant activity than Trolox across all concentrations, whereas there are varying results on E-UAE and E-MAE. In the FRAP test, the EC_50_ values of C3, C4, and E-UAE are lower than the EC_50_ value of Trolox. Similarly, in the DPPH assay, the EC_50_ values of C3 and C4 are lower than the EC_50_ value of Trolox, indicating the effectivity and efficiency of these compounds as antioxidants.

#### 2.3.2. Antidiabetes: α-Glucosidase and Acarbose Control

**Figure 2:**
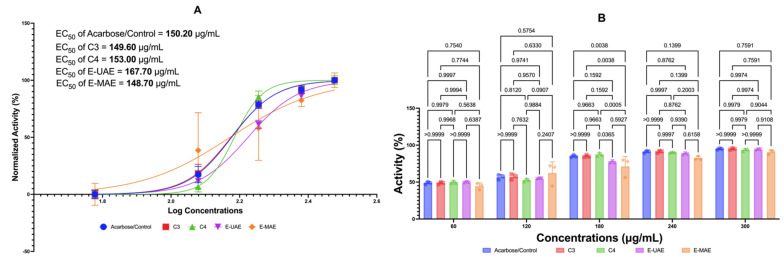
Antidiabetic potential of *E. acoroides* through α-glucosidase inhibition. (**A**) EC_50_ of α- glucosidase inhibition activity. (**B**) Difference in antidiabetic activity in a-glucosidase inhibition activity based on two-way ANOVA. C3, astaxanthin; C4, canthaxanthin; E-UAE: *E. acoroides*—ultrasound-assisted extraction; E-MAE: *E. acoroides*—microwave-assisted extraction.

Figure 2 displays the inhibitory effects of C3, C4, E-UAE, and E-MAE on α-glucosidase. All these compounds suppressed the activity of α-glucosidase similar to acarbose at concentrations of 180, 240, and 300 μg/mL, except for E-MAE, where its effectivity as an antidiabetic agent is similar to acarbose starting at the concentration of 240 μg/mL. Acarbose was used as the control antidiabetic agent in this study. The EC_50_ values for acarbose, C3, C4, E-UAE, and E-MAE are 149.60 μg/mL, 153.00 μg/mL, 167.70 μg/mL, and 148.70 μg/mL, respectively. The EC_50_ values for C3 and E-MAE, specifically, are lower than the EC_50_ value of acarbose, indicating the effectivity and efficiency of these compounds as antidiabetic agents.

#### 2.3.3. Antiobesity: Lipase and In Vitro MTT Assay 3T3-L1 Control Orlistat and Simvastatin

**Figure 3:**
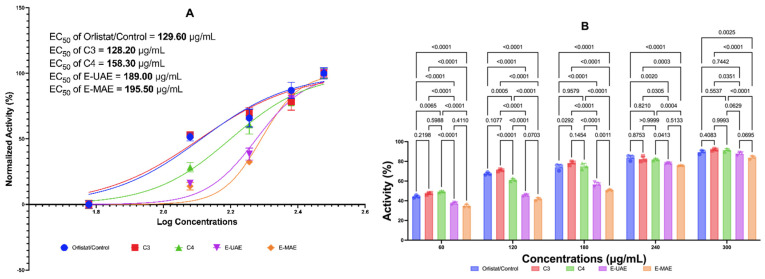
Antiobesity potential of *E. acoroides* through lipase inhibition. (**A**) EC_50_ of lipase inhibition activity. (**B**) Difference in antiobesity activity in lipase inhibition based on two-way ANOVA. C3, astaxanthin; C4, canthaxanthin; E-UAE: *E. acoroides*—ultrasound-assisted extraction; E-MAE: *E. acoroides*—microwave-assisted extraction.

Figure 3 displays the inhibitory activities of C3, C4, E-UAE, and E-MAE against pancreatic lipase. At concentrations of 60 μg/mL, C4 has a better antiobesity activity than orlistat, while C3 has a similar activity to orlistat. At 120 μg/mL, only C3 has a similar activity to orlistat. At higher concentrations (180, 240, and 300 μg/mL), C3 and C4 exhibited similar antiobesity activity to orlistat, suggesting they could be natural substitutes for the drug. The EC_50_ values for orlistat, C3, C4, E-UAE, and E-MAE are 129.60 μg/mL, 128.20 μg/mL, 158.30 μg/mL, 189.00 μg/mL, and 195.50 μg/mL, respectively. The EC_50_ single compound or C3 was lower than orlistat control, this suggests that C3 exhibits promising inhibitory effects on pancreatic lipase comparable to orlistat.

**Figure 4:**
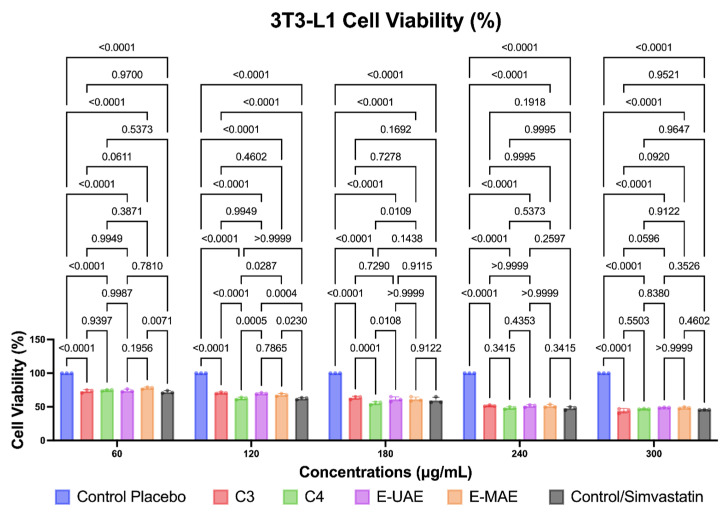
Difference in antiobesity activity in 3T3-L1 preadipocytes cell inhibition, based on two-way ANOVA. C3, astaxanthin; C4, canthaxanthin; E-UAE: *E. acoroides*—ultrasound-assisted extraction; E-MAE: *E. acoroides*—microwave-assisted extraction.

Compounds C3, C4, E-UAE, and E-MAE also decreased the number of viable 3T3-L1 cells, as illustrated in Figure 4. The reduction in viable cells was observed at 60, 120, 180, 240, and 300 μg/mL concentrations for C3, C4, E-UAE, and E-MAE, respectively. Simvastatin was used as the control in this study. The reduction in viable cells was more significant with C3, C4, E-UAE, and E-MAE compared to the placebo, and the effects were comparable to those of simvastatin. However, at concentrations of 240 μg/mL and higher, the interventions resulted in a deleterious effect on cell viability, reducing it by almost 50%. Despite this, the results were still less effective than those achieved with simvastatin, a widely available drug known for lowering blood cholesterol.

#### 2.3.4. miR-21/132 Expressions

**Figure 5:**
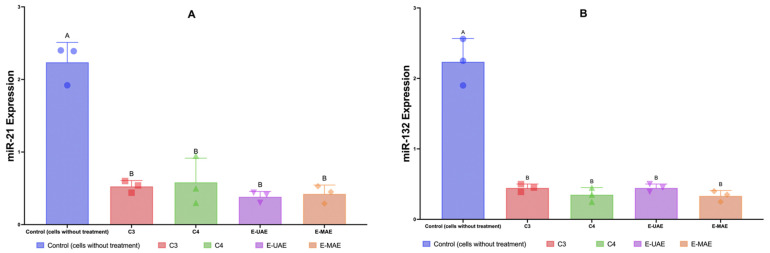
Downregulation of microRNA-21 (**A**) and microRNA-132 (**B**). Letters (A,B) denote significant differences (*p* < 0.05; 95% CI; one-way ANOVA). C3, astaxanthin; C4, canthaxanthin; E-UAE: *E. acoroides*—ultrasound-assisted extraction; E-MAE: *E. acoroides*—microwave-assisted extraction.

The results compare the expressions of microRNA-21 and microRNA-132 between a control group (cells without treatment) and groups treated with C3, C4, E-UAE, and E-MAE (Figure 5). For microRNA-21, the control group shows a high level of expression, indicated by the height of the blue bar (Figure 5). Treatments with E-UAE, E-MAE, C3, and C4 significantly reduce the expression microRNA-21 compared to the control. E-UAE shows a slightly greater reduction compared to E-MAE, as indicated by the shorter purple bar compared to the orange bar (Figure 5). Regarding microRNA-132, the control group again has a high expression level. Similar to microRNA-21, treatments with C3, C4, E-UAE, and E-MAE result in a reduction in microRNA-132 expression. The statistical significance of the differences between the control and treatment groups is indicated above the bars.

## 3. Discussion

Ultrasound-assisted extraction (UAE) and microwave-assisted extraction (MAE) are innovative techniques that have gained significant attention in extraction processes. UAE uses ultrasound energy and solvents to extract target compounds from various plant matrices [15]. Conversely, MAE utilizes microwave energy to facilitate the extraction of bioactive compounds [16]. Both methods have been recognized for their efficiency, reduced environmental impact, high extraction yields, and shorter extraction times, compared to traditional methods [16]. A study employed a combination of enzyme-assisted extraction (EAE) and UAE to extract flavonoids from cashew nut testa, showcasing the effectiveness of ultrasound in extraction processes [17]. Additionally, another study highlighted that UAE resulted in higher yields of crude extract from *Eugenia* spp., further emphasizing the efficacy of ultrasound-assisted extraction [18]. Furthermore, a study optimized UAE for extracting phenolic compounds from apple tree leaves, demonstrating that the optimization of UAE parameters can enhance extraction yields while preserving the biological activity of the extract [19]. This is in line with recent data in this study and it appears that UAE is a green extraction method that also has the potential to extract carotenoid compounds in seagrass *E. acoroides*.

Microwave-assisted extraction (MAE) has been reported to significantly enhance the extraction rate and increase the extraction yield compared to traditional methods [20,21]. It offers advantages such as shorter extraction times, lower solvent consumption, and higher extraction rates, making it a promising approach for extracting plant constituents [22]. Studies have shown that MAE significantly reduces extraction time compared to conventional methods such as Soxhlet extraction, with extraction times typically less than 30 min [23]. MAE has been used to produce extracts of superior quality at a lower cost due to its higher extraction rate [21,22]. Furthermore, MAE has been successfully applied to extract various compounds from different sources, such as essential oils, antioxidants, phenolic compounds, and natural antioxidants [22,24,25,26]. The efficiency of MAE has been demonstrated in the extraction of a wide range of compounds, including phenolic compounds, flavonoids, antioxidants, and alkaloids [27], and it turns out that it can also elucidate carotenoid compounds, as demonstrated in this study. The data are presented in Table 1 and Table 2.

**Figure 6:**
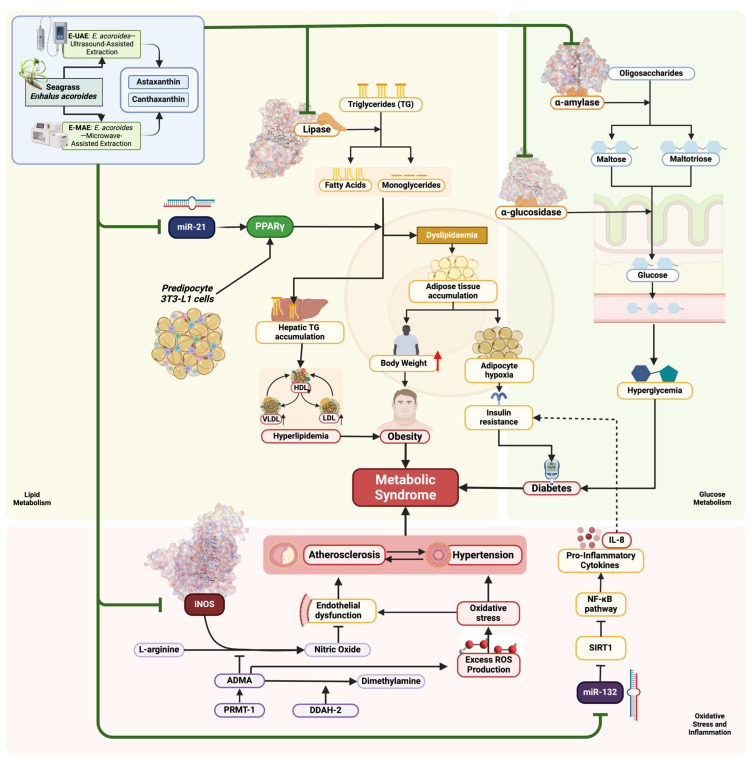
Biomechanism of astaxanthin, canthaxanthin, E-UAE, and E-MAE as antioxidants, antidiabetic, and antiobesity compounds.

We found that C3, C4, E-UAE, and E-MAE demonstrated significant potential in inhibiting several targets: iNOS (3E7G) in terms of antioxidative activity; lipase (1LPB) in terms of antiobesity activity; and α-glucosidase (3L4Y) and α-amylase (2QV4) in terms of antidiabetic activities. These compounds also inhibited the expression of microRNA-21, acting as a regulator of lipid metabolism, and microRNA-132, demonstrating anti-inflammatory properties in relation to their antidiabetic effects. The biomechanism by which these compounds potentially affect each corresponding metabolic cascade and body physiology, specifically their antidiabetic, antiobesity, and antioxidative effects, are visually described in Figure 6.

Antioxidants play a crucial role in combating oxidative stress and inflammation in various physiological conditions. The inhibition of inducible nitric oxide synthase (iNOS) expression and the reduction in nitric oxide (NO) concentration have enhanced antioxidant capacity and reduced oxidative stress injury [28]. Synthetic iNOS inhibitors, classified based on their chemical structures, offer potential therapeutic benefits in conditions such as Alzheimer’s disease [29]. Furthermore, compounds such as pentoxifylline have decreased iNOS expression, particularly under hypoxic conditions, highlighting their potential in modulating oxidative stress [30]. Electrolyzed Reduced Water (ERW) has been found to inhibit oxidative stress by restoring the antioxidant capacity of enzymes such as superoxide dismutase and catalase, thereby impacting the NF-κB/iNOS pathway [31]. Additionally, iNOS inhibitors have been extensively studied for their roles in various conditions, such as multiple sclerosis, inflammatory bowel diseases, Alzheimer’s disease, and rheumatoid arthritis [32].

Lipase inhibition is a crucial mechanism that can have significant implications in various health aspects, including antioxidants, antidiabetes, and antiobesity effects. Several studies have explored the potential of natural compounds in inhibiting lipase activity and their subsequent effects on health. For instance, a study investigated the inhibitory activity of herbal medicines on key enzymes related to carbohydrate and lipid digestion, highlighting the potential of plant extracts in inhibiting enzymes such as pancreatic lipase [33]. Similarly, research by Wiyono et al. (2022) focused on the beneficial properties of Javanese *Tamarindus indica* leaves in inhibiting lipase enzymes, indicating the potential of natural sources in combating obesity [34]. Moreover, studies emphasized the antiobesity and antioxidant potentials of selected medicinal plants and plant extracts, showcasing their ability to inhibit pancreatic lipase and suggesting their application in managing obesity and related conditions [35,36]. Additionally, the work by Krishnamurthy et al. (2020) highlighted the antiobesity and antioxidant activities of *Hibiscus sabdariffa* extract through the inhibition of pancreatic lipase and alpha-glucosidase, further underlining the role of natural compounds in promoting health [37]. Furthermore, investigations by Noorolahi et al. (2020) on the tannin fraction of pistachio green-hull extract and by Aabideen et al. (2020) on *Taraxacum officinale* provided insights into the pancreatic lipase inhibitory and antioxidant activities of plant extracts, suggesting their potential as functional foods for managing obesity [38,39]. These studies collectively demonstrate the promising role of natural compounds in inhibiting lipase activity, which can contribute to antioxidant, antidiabetes, and antiobesity effects.

α-Glucosidase inhibitors play a crucial role in managing conditions such as diabetes and obesity by slowing down the absorption of glucose in the body. These inhibitors work by targeting the α-glucosidase enzyme, which is responsible for breaking down complex carbohydrates into glucose. By inhibiting this enzyme, the absorption of glucose is delayed, leading to lower blood glucose levels [40]. Various natural compounds have been studied for their α-glucosidase inhibitory effects. For instance, compounds derived from plants such as *Garcinia mangostana*, African black velvet tamarind, and *Rhizophora mucronata*, have shown antioxidant properties and the ability to inhibit α-glucosidase, thus potentially aiding in managing diabetes [41,42]. Additionally, compounds from sources such as mangrove endophytic fungi and marine sponge-derived fungi have demonstrated significant α-glucosidase inhibitory activity, highlighting their potential as natural inhibitors for managing diabetes [43,44]. Moreover, studies have identified specific compounds, such as diketopiperazine alkaloids and alkaloids from fungal sources, which exhibit potent α-glucosidase inhibitory effects, indicating their potential as therapeutic agents for diabetes [45,46]. Furthermore, essential oils from plants such as Pelargonium graveolens have shown promise in controlling postprandial hyperglycemia through α-glucosidase enzyme inhibition, suggesting their utility in diabetes management [47].

α-Amylase inhibition is a crucial mechanism in the management of conditions such as diabetes and obesity. Several studies have explored the potential of natural compounds in inhibiting α-amylase activity, which is essential for controlling postprandial hyperglycemia and managing type 2 diabetes. For instance, *Costus igneus*, *Gymnema sylvestre*, and *Ocimum sanctum* have demonstrated significant α-amylase inhibitory activity [48]. Moreover, the inhibition of α-amylase is considered an effective therapeutic approach against chronic type 2 diabetes mellitus [49]. Compounds such as 1,2,3-triazole derivatives have shown significant antidiabetic activity by inhibiting α-amylase [50]. The α-amylase inhibitory potential of indigenous plants has also been investigated, highlighting the importance of inhibiting carbohydrate-hydrolyzing enzymes for managing diabetes [51].

MicroRNAs (miRNAs) play a significant role in obesity, particularly miR-21 and miR-132. These miRNAs are implicated in regulating adipogenesis, lipid metabolism, fat accumulation, inflammation, and insulin resistance in the context of obesity [52,53,54,55,56,57]. Studies have shown that miR-21 and miR-132 are associated with adipocyte proliferation, differentiation, and metabolic syndrome, and their dysregulation is linked to obesity-related disorders such as type 2 diabetes mellitus (T2DM) [52,53,54,55,56]. Additionally, miR-132 has been found to modulate glucose-stimulated insulin secretion and improve beta cell function in obesity models [57,58].

As with obesity, miR-21 and miR-132 have been identified as significant players in the context of diabetes. Studies have highlighted the potential of miR-21 and miR-132 as biomarkers for diabetic dyslipidemia [59]. These microRNAs have been consistently reported in various tissues and blood samples of individuals with type 2 diabetes [60]. In diabetic encephalopathy, decreased levels of miR-132 have been associated with the dysregulation of the GSK-3β/Tau pathway, contributing to diabetic complications [61]. In the context of obesity, the peroxisome proliferator-activated receptor gamma (PPAR-γ) and microRNA-21 (miR-21) play significant roles. PPAR-γ is a key regulator of adipogenesis and fat metabolism [62]. Studies have shown that PPAR-γ is associated with weight loss after interventions such as sleeve gastrectomy and is upregulated in the adipose tissue of obese individuals [62,63]. Conversely, miR-21 has been linked to obesity independently of glycemic state and is involved in adipose tissue functionality by regulating various genes and processes related to obesity [52]. Additionally, miR-21 has been shown to participate in liver lipid metabolism and contribute to non-alcoholic steatohepatitis (NASH) through PPAR-α [64]. Furthermore, miR-21 has been found to be upregulated in subcutaneous adipose tissue in human obesity and positively correlated with the body mass index (BMI) [65]. Moreover, miR-21 has been associated with adipose tissue inflammation and insulin resistance by targeting PPAR-γ [64]. Specifically, miR-21 has been identified as a key therapeutic target for renal injury in type 2 diabetes [66]. This indicates a complex interplay between miR-21, PPAR-γ, and obesity-related metabolic alterations.

The roles of miR-132 extend beyond diabetes, as it has been shown to inhibit high glucose-induced vascular smooth muscle cell proliferation and migration, suggesting a potential therapeutic approach for cardiovascular disease in diabetes [67]. Additionally, engineered exosomes derived from miR-132-overexpressing adipose stem cells have demonstrated effectiveness in promoting diabetic wound healing due to their anti-inflammatory and angiogenic properties [68]. Furthermore, miR-132 has been associated with chronic wound healing and has shown therapeutic potential in various types of chronic wounds beyond diabetes [69]. In pancreatic beta cells, miR-132 controls proliferation and survival through the Pten/Akt/Foxo3 signaling pathway, indicating its involvement in maintaining beta cell function under metabolic stress conditions such as obesity-induced diabetes [70].

## 4. Materials and Methods

### 4.1. Preparation and Extraction of E. acoroides

The sample collection was carried out with formal authorization from both the local authorities and the proprietor of the local community. The seagrass species, *E. acoroides*, was collected from the waters of Manado Bay in Manado City, situated in the North Sulawesi Province of Indonesia, with precise coordinates of 1°43′11.5″ N 124°48′02.7″ E. The specimens were botanically identified and authenticated by an experienced biologist and the authors, adhering to the guidelines provided by the National Center for Biotechnology Information (NCBI) Taxonomy ID NCBI:txid55455. These specimens belong to the taxonomic classification of cellular organisms, specifically within the categories *Eukaryota*, *Viridiplantae*, *Streptophyta*, *Streptophytina*, *Embryophyta*, *Tracheophyta*, *Euphyllophyta*, *Spermatophyta*, *Magnoliopsida*, *Mesangiospermae*, *Liliopsida*, *Alismatales*, *Hydrocharitaceae*, and *Enhalus*. The primary purpose of collecting these specimens was to document them for future reference.

The methodologies employed in this study adhered strictly to the established guidelines and regulations for in vitro and seagrass research. The seagrass species *E. acoroides* underwent a comprehensive purification process to remove any soil particles by repeatedly cleaning using distilled water. Following purification, the material was securely wrapped and dried in a Memmert Incubator IN55 oven (Schwabach, Germany) at a constant temperature of 60 °C for 72 h. Once dried, the seagrass was cut into small pieces and ground into simplicia powder using a Cosmos blender 2 L ReBlend High-Speed Hand Blender (Tangerang, Indonesia) to reduce sample size and create a coarse simplicia powder. The simplicia powder was then subjected to extraction using green methods, specifically ultrasound-assisted extraction (UAE) and microwave-assisted extraction (MAE), and the results were compared.

For the UAE method, 200 g of *E. acoroides* simplicia powder were subjected to sonication for 30 min at 40 °C using a Branson 2510 ultrasound sonicator (St. Louis, MO, USA) with a power output of 400 W. The sonication process was performed in the presence of two liters of deionized water. The resulting mixture underwent filtration, re-extraction, and concentration using a rotary evaporator at 100 °C, yielding a dense extract labeled as *E. acoroides* ultrasound-assisted extraction (E-UAE). This extract was then preserved in aluminum foil for future analyses.

For the MAE method, 200 g of *E. acoroides* simplicia powder were combined with two liters of deionized water as the solvent. The extraction was carried out in a beaker, used as the microwave extraction device. The process was conducted at a microwave power of 165 W for 9 min using a closed microwave system, specifically the CEM Corporation’s Discover-SP model (Matthews, NC, USA), with the temperature maintained below 110 °C. The resulting extract was stored in a dark glass container at −20 °C until further analysis. This approach aligns with methods used in similar research [71], resulting in the acquisition of *E. acoroides* microwave-assisted extract (E-MAE). For better understanding, Graphical Abstract illustrates the various phases of the study.

### 4.2. Carotenoid Identification and Analysis of E. acoroides via UHPLC-ESI-MS/MS

In the LC-MS analysis, a solution of *E. acoroides* extract was combined with a dichloromethane (DCM)/methanol (MeOH) solution in a 50:50 ratio. The mixture was filtered using a 0.22 μm nylon filter to remove impurities. The clarified solution was then introduced into the UHPLC system at a concentration of 1 mg/mL. The preparation of the standard stock solution followed the protocol established by Balasubramaniam et al. (2020) [72]. A stock solution with a concentration of 10 mg/mL was prepared by dissolving the analyte in a DCM/MeOH (50:50) solution. This solution was stored at −20 °C, protected from light. These stock solutions were used for spiking at various levels to validate the method. Only primary grade standards with a purity of approximately 99.5% were used for LC-MS/MS analysis. The chemical standards used in this study—β-carotene, lutein, lycopene, β-cryptoxanthin, and zeaxanthin—were obtained from Sigma-Aldrich^®^ (St. Louis, MO, USA).

The modified protocol described by Balasubrama-niam et al. (2020) [72] was employed to identify the carotenoids, which was performed using the UHPLC-ESI/HRMS/MSn system. The Dionex UHPLC Ultimate 3000 (Thermo Fisher Scientific, Waltham, MA, USA) and the Q Exactive Hybrid Quadrupole-Orbitrap Mass Spectrometer (Thermo Fisher Scientific, Waltham, MA, USA) are the components of this system. The column utilized was a C18 reversed-phase column from Acquity UPLC BEH C18, with dimensions of 50 mm × 2.1 mm and a particle size of 1.7 μm, which was compatible with MS. The autosampler was configured to operate at a temperature of 4 °C. The column was loaded with a 3 μL sample volume and maintained at 35 °C. At a flow rate of 0.3 mL/min, the gradient program utilized two mobile phases: water with 0.1% formic acid (A) and acetonitrile (ACN) with 0.1% formic acid (B). The gradient was as follows: B increased from 5% to 99% from 2 to 20 min, from 5% to 99% from 20 to 25 min, and from 5% to 5% from 25 to 30 min.

Both negative and positive ion electrospray ionization (ESI) modes were utilized for the MS analysis. The mass spectrometer operated within a scanning range of 100–1000 *m*/*z* and a resolving power of 140,000 FWHM. The sheath and auxiliary gas flow rates were set to 35 and 12 arbitrary units, respectively, with the capillary temperature maintained at 320 °C. The spray voltage was 3.7 kV, and the S-lens voltage was 55 V for both positive and negative ionization modes. Data processing was carried out using Xcalibur 2.1.0 (Thermo Fisher Scientific, Waltham, MA, USA) after the MS/MS spectra were acquired with a collision energy of 35 V. For a comprehensive understanding of peak identification and validation of the UHPLC-MS/MS method, please refer to the 2020 work of Balasubramaniam et al. [72].

### 4.3. Evaluation of In Silico Study

#### 4.3.1. Predicting the Activities of Bioactive Compounds, Analyzing Toxicity, and Assessing Drug-Likeness

The bioactivity of the compounds derived from *E. acoroides* was evaluated using the WAY2DRUG PASS prediction tool (https://www.way2drug.com/PassOnline/predict.php, accessed on 20 July 2024). This tool was employed to assess the compounds’ ability to selectively target malignant melanoma tumors. The analysis included performing a Structure–Activity Relationship (SAR) analysis to compare the input compounds with well-established bioactive compounds. The Pa value, a predictive score provided by the online tool, indicates the potency of a compound [73]. A Pa value exceeding 0.7 suggests potential bioactivity, such as antiobesity and lipid metabolism regulation, as it closely resembles known compounds in the database. Higher Pa values indicate greater precision in predictions. This study specifically investigated compounds with Pa values exceeding 0.4. Additionally, toxicity and drug-likeness analyses were conducted to evaluate the pharmacokinetic properties and potential adverse effects of the compounds. The drug similarity properties of each ligand were evaluated using Lipinski’s Rule of Five (Ro5). These analyses were conducted using the Protox II database (https://tox-new.charite.de/protox_II/index.php?site=compound_input, accessed on 20 July 2024) and the ADMETLab 2.0 database (https://admetmesh.scbdd.com/service/evaluation/index, accessed on 20 July 2024). The SMILES notation for each compound was used as input for these analyses. The SMILES representations were obtained from PubChem (https://pubchem.ncbi.nlm.nih.gov, accessed on 20 July 2024), with the corresponding data provided in Supplementary Data Table S2.

#### 4.3.2. Simulated Molecular Docking

The docking simulation was performed using CB-Dock2, an advanced version of the CB-Dock server. CB-Dock2 utilizes cavity-detection-guided blind docking to investigate interactions between proteins and ligands. This method integrates cavity identification, docking, and homologous template fitting, as detailed in previous studies [74]. CB-Dock2 streamlines the docking process by identifying binding locations, calculating their positions and sizes, adjusting the docking area based on the molecules being studied, and performing molecular docking. The CurPocket method employs curvature-based cavity detection to predict the binding sites of target proteins, while CB-Dock2 identifies the binding positions of query ligands. For a comprehensive methodology, refer to the publication by Liu and Cao (2024) [74].

The receptors with the highest centrality, associated with their respective signaling pathways, were selected for further molecular docking investigations. The genetic material or proteins used in this study were iNOS (PDB ID: 3E7G), Lipase (PDB ID: 1LPB), α-Glucosidase (PDB ID: 3L4Y), and α-Amylase (PDB ID: 2QV4). Before docking, the CB-Dock2 server automatically removed water molecules and other heteroatoms from the protein structures. All proteins served as receptors or targets for ligand binding. The protein structures in .pdb format were obtained from the RSCB Protein Data Bank (https://www.rcsb.org; accessed on 21 July 2024). The ligands were sourced from PubChem in .sdf format (https://pubchem.ncbi.nlm.nih.gov; accessed on 21 July 2024). CB-Dock2 enhances docking precision through the integration of cavity detection, docking, and homologous template fitting. This approach allows for the prediction of binding sites and their affinities, which is advantageous for drug discovery. The affinity (ΔG) value is used to assess the effectiveness of the ligand or target compound and to compare it with a control or standard drug.

### 4.4. Evaluation of In Vitro Study

#### 4.4.1. Antioxidants Evaluation through DPPH Radical Scavenging Activities and FRAP Assay

The antioxidant activity of seagrass extracts was evaluated using two distinct assays: DPPH radical scavenging activity and the FRAP test, following methodologies from previous studies [75,76]. Seagrass extract samples were introduced into testing vials containing 3 mL of DPPH reagent, with concentrations set at 60, 120, 180, 240, and 300 μg/mL. The DPPH-sample mixtures were then refrigerated for 30 min at ambient temperature. The change in the DPPH concentration was determined by measuring the absorbance at a wavelength of 517 nm. The percentage of DPPH inhibition was calculated using the following method. A stock solution was mixed with 60 mL of ethanol to produce a working solution with an absorbance of 0.706 at 734 nm. For the test, a fresh solution was prepared with varying concentrations of 60, 120, 180, 240, and 300 μg/mL. The samples were diluted with 1 mL of the ABTS working solution, and their absorbance was measured at a wavelength of 734 nm after 7 min. The formula used to compute the percentage of DPPH inhibition is as follows:

$$\mathrm{Inhibition}\;\mathrm{Activity}\;(\%)=\frac{A0-A1}{A0}\times 100\%$$

A0 = absorbance of blank; A1 = absorbance of standard or sample.

The FRAP test was performed in accordance with the methodology outlined by Youn et al. (2019) [77]. The FRAP reagent was produced by combining 1 mL of 10 millimolar (mM) TPTZ in 40 mM hydrochloric acid, 20 mM ferric (III) chloride, and 1 mL of 300 mM sodium acetate buffer at pH 3.6. A water bath was employed to maintain the FRAP reagent mixture at 37 °C. Subsequently, 1 mL of the FRAP reagent was combined with seagrass extract samples of varying concentrations. In order to ascertain the antioxidant activity, the absorbance of the mixture was promptly measured at a wavelength of 593 nm using the equation provided:

$$\mathrm{FRAP}\;(\%)=\left[\frac{Ac-Ab}{Ac-Ab}\right]\times 2$$

Ac = absorbance of positive control; Ab = absorbance of the blank.

To ensure the reliability of the DPPH and FRAP assay results, each sample was tested in triplicate (*n* = 3). Trolox (C_14_H_18_O_4_; PubChem CID: 40634), a well-known antioxidant compound, was used as the positive control in both assays. The antioxidant activity of the samples and Trolox was quantified using the half-maximal effective concentration (EC_50_), which indicates the concentration of a sample required to achieve a 50% reduction in the initial radical concentration.

#### 4.4.2. Antidiabetes Evaluation through α-Glucosidase Inhibition

The methodology outlined in prior literature was employed to evaluate the inhibitory activity of α-glucosidase [78,79,80]. A concentration of 1.52 IU/mL was achieved by preparing a solution containing the enzyme (76 IU, 1 mg) and a phosphate buffer (50 mL, pH 6.9). The following substances were sequentially added to the reaction tube: 0.35 mL of sucrose (65 mM concentration), a maltose solution (65 mM concentration), and samples labeled C3, C4, E-UAE, and E-MAE, at concentrations of 60, 120, 180, 240, and 300 μg/mL, respectively. After homogenization, 0.2 mL of an α-glucosidase solution (1.52 IU/mL) was introduced into each tube. The tubes were then incubated at 37 °C for 20 min. After the incubation period, the enzyme was deactivated by heating the tubes in a water bath at 100 °C for 2 min. Acarbose served as the positive control. To improve the color, 0.2 mL of a testing solution was combined with 3 mL of a color reagent, and the mixture was heated to 37 °C for 5 min. The solution’s absorption was analyzed at a wavelength of 505 nm. The inhibitory activity was determined by the quantity of glucose liberated during the reaction [80].

#### 4.4.3. Antiobesity Evaluation: Lipase Inhibition and an In Vitro MTT Assay with the 3T3-L1 Cell Line

Pure pig pancreatic lipase (PPL) was initially dispersed in a 50 mM phosphate buffer (pH 7) at a concentration of 1 mg/mL. The solution was then centrifuged at 12,000× *g* to remove any insoluble substances. To create an enzyme stock solution with a concentration of 0.1 mg/mL, the supernatant was diluted tenfold with the buffer solution. Previous research methods were used to assess the lipase inhibitory capacity. In a clear 96-well microplate, 20 µL of 10 mM p-nitrophenyl butyrate (pNPB) in buffer was combined with 100 µL of the C3, C4, E-UAE, and E-MAE samples. The mixture was incubated at 37 °C for 10 min. The results were compared to those obtained with orlistat (C_29_H_53_NO_5_, PubChem CID: 3034010), a widely recognized lipase inhibitor. Measurements were performed at a wavelength of 405 nm using a DR-200Bc ELISA microplate reader. The activity unit was determined by measuring the reaction rate of 1 mole of p-nitrophenol (4-nitrophenol, C_6_H_5_NO_3_) per minute at 37 °C. To evaluate the level of lipase inhibition, the activity of PPL was reduced by a specific amount in the test mixture. Each sample was tested in triplicate (*n* = 3) to ensure the reliability and accuracy of the study’s findings. The inhibitory data were calculated using the equation described in previous research [78,79].

$$\mathrm{Inhibition}\;\mathrm{of}\;\mathrm{Lipase}\;(\%)=100-\frac{B-Bc}{A-Ac}$$

A = activity without inhibitor; B = activity with inhibitor; Ac = negative control (−) without inhibitor; Bc = negative control (−) with inhibitor.

For the first 24 h, 3T3-L1 preadipocyte cells were cultured in DMEM with 10% FBS at 37 °C in a 5% CO_2_ atmosphere. The cells were seeded in 6-well plates at a density of 1 × 10^5^ cells per well. Modifications to the methodologies employed in this investigation were based on prior studies [81,82]. To achieve adipocyte differentiation, the 3T3-L1 cells were subsequently subjected to a differentiation medium consisting of DMEM with 10% FBS, 0.5 mM 3-isobutyl-1-methylxanthine (IBMX), 1 μM dexamethasone, and 10 μg/mL insulin. To maintain the adipocyte characteristics, the cells were incubated with a fresh insulin medium (DMEM with 10% FBS and 10 μg/mL insulin) for three days on day five. The effects of simvastatin (60, 120, 180, 240, and 300 μg/mL) and C3, C4, E-UAE, and E-MAE (60, 120, 180, 240, and 300 μg/mL) were assessed in triplicate in the differentiation medium. The viability of the 3T3-L1 preadipocyte cells (American Type Culture Collection, Manassas, VA, USA) was evaluated using the MTT assay. Initially, the cells were cultured in a 96-well plate at a density of 5 × 10^3^ cells per well in DMEM with 10% FBS at 37 °C in a 5% CO_2_ atmosphere for 24 h. Following this incubation, the cells were treated with different extract dosages and simvastatin for 72 h. Subsequently, 100 μL of the MTT solution (5 mg/mL) was added to each well and incubated at 37 °C for an additional four hours. After incubation, MTT–formazan crystals in live cells were dissolved in 100 μL of DMSO, and the absorbance was measured at 540 nm. The percentage of cell viability was determined by comparing the absorbance of the viable cells in the treatment wells to those in the control wells.

#### 4.4.4. The miRNA Expression of miR-21/132

In order to extract total RNA from epididymal adipose tissue, the TRIzol reagent (GeneAll Biotechnology, Seoul, Republic of Korea) was used according to the manufacturer’s instructions. cDNA was synthesized from the total RNA using a Moloney Murine Leukemia Virus (M-MLV) Reverse Transcriptase kit (Bioneer Co., Daejeon, Republic of Korea). Real-time quantitative polymerase chain reaction (qPCR) was conducted with the Rotor-Gene 3000 instrument (Corbett Research, Mortlake, NSW, Australia) and AccuPower 2X Greenstar qPCR MasterMix (Bioneer Co., Daejeon, Republic of Korea). Data were analyzed using the 2^−ΔΔCt^ method, with β-actin serving as the reference gene for normalization. For miRNA expression analysis, cDNA was produced using a miRNA cDNA Synthesis Kit with Poly (A) Polymerase Tailing (ABM Inc., Richmond, BC, Canada). The synthetically generated cDNA was amplified using EvaGreen miRNA qPCR Master Mix (ABM Inc.). Specific primers for miR-21, miR-132, and U6 (ABM Inc.) were employed to quantify miRNAs. Real-time qPCR amplification was performed with the Rotor-Gene 3000 (Corbett Research). The expression levels of miR-21 and miR-132 were normalized to U6 snRNA and calculated using the 2^−ΔΔCt^ technique.

### 4.5. Data Management and Analysis

The data were analyzed using the MacBook version of GraphPad Prism Premium 10 software from GraphPad Software, Inc. (San Diego, CA, USA). The Shapiro–Wilk test was implemented to evaluate the distribution of the data. If the data followed a normal distribution with a significance level of less than 0.05, mean differences between treatment groups were analyzed using either a one-way or two-way ANOVA test. A two-way ANOVA was used to determine the statistical significance of antioxidant activity (measured by FRAP and DPPH), lipase activity, α-glucosidase inhibition, and the cytotoxicity of the 3T3-L1 cell line, at a 95% confidence interval. A one-way ANOVA was applied to analyze differences in the carotenoid concentration between E-UAE and E-MAE, as well as miR-21 and miR-132 expression. If the data did not adhere to a normal distribution, the Kruskal–Wallis test was performed instead. The EC_50_ values for antioxidant activity (FRAP, DPPH), lipase, and α-glucosidase inhibition activities at an effective concentration of 50% were analyzed using the non-linear regression function in the GraphPad Prism Premium statistical analysis package (log(inhibitor) vs. normalized response variable slope).

## 5. Conclusions

The findings from this study underscore the significant presence of carotenoids in *E. acoroides*, with fucoxanthin, lutein, and β-carotene identified as the predominant compounds. Through comprehensive mass spectrometry and chromatography techniques, the green extraction methods utilized—ultrasound-assisted extraction (E-UAE) and microwave-assisted extraction (E-MAE)—proved effective, with E-UAE yielding higher carotenoid levels. Additionally, in silico and in vitro analyses revealed that these carotenoids exhibit substantial antioxidant, antidiabetic, and antiobesity properties. The compounds C3, C4, and C7 demonstrated notable interactions with key metabolic proteins and microRNAs, further validating their potential therapeutic benefits.

Considering the promising bioactive properties identified in *E. acoroides* carotenoids, future research should focus on detailed in vivo studies to confirm their efficacy and safety as therapeutic agents. The exploration of optimized extraction techniques can enhance yield and purity, making these compounds more viable for clinical applications. Additionally, further investigation into the molecular mechanisms underpinning their bioactivities could lead to the development of novel treatments for metabolic disorders. Overall, this research lays the groundwork for leveraging *E. acoroides* carotenoids in the pharmaceutical and nutraceutical industries, potentially offering natural alternatives for managing oxidative stress, diabetes, and obesity.

## Figures and Tables

**Table 1:** Measured theoretical and accurate masses of the targeted carotenoid in *E. acoroides* by Orbitrap-MS.

Observed Compounds	Selected Ion	Observed MW	Molecular Formula	T^R^ (min)	PubChem CID	CAS Number
Fucoxanthin	[M + H–H_2_O]^+^	658.8779	C_42_H_58_O_6_	16.19	5281239	3351-86-8
Lutein	[M + H–H_2_O]^+^	568.4239	C_40_H_56_O_2_	20.22	5281243	127-40-2
Astaxanthin	[M + H]^+^	596.5900	C_40_H_52_O_4_	18.99	5281224	472-61-7
Canthaxanthin	[M + H]^+^	564.7000	C_40_H_52_O_2_	21.15	5281227	514-78-3
Zeaxanthin	[M]^+^	568.7209	C_40_H_56_O_2_	20.09	5280899	144-68-3
β-Cryptoxanthin	[M]^+^	552.8838	C_40_H_56_O	22.49	5281235	472-70-8

T^R^: retention time (minutes); MW: molecular weight.

**Table 2:** Carotenoid observed in dry weight (mg/100 g) of *E. acoroides* via UHPLC-ESI-MS analysis.

Samples	Fucoxanthin	Astaxanthin	Zeaxanthin	Lutein	β-Carotene	β-Cryptoxanthin	Canthaxanthin
E-UAE	7.65 ± 1.00 ^a^	6.80 ± 1.03 ^a^	6.35 ± 0.33 ^a^	9.73 ± 0.43 ^a^	8.58 ± 0.54 ^a^	2.61 ± 0.63 ^a^	3.51 ± 1.04 ^a^
E-MAE	6.08 ± 0.64 ^b^	5.18 ± 0.56 ^b^	3.66 ± 0.26 ^b^	7.69 ± 0.87 ^b^	6.57 ± 0.52 ^b^	4.49 ± 0.16 ^b^	2.04 ± 0.13 ^a^

E-UAE: *E. acoroides*—ultrasound-assisted extraction; E-MAE: *E. acoroides*—microwave-assisted extraction. Values are presented as means ± SD of triplicate analysis (*n* = 3). Letters (a,b) denote significant differences (*p* < 0.05; 95% CI; two-way ANOVA) between the mean values within the same column.

**Table 3:** Evaluating carotenoid in *E. acoroides* potential for antidiabetes and antiobesity based on structure–activity relationship (SAR) predictions via Pa score, toxicity prediction, and drug-likeness analysis.

Code	Pa Score *	Toxicity Model Computation Analysis **		Drug-Likeness ***		
	>0.4	Predicted LD_50_ (mg/kg BW)	Toxicity Class	Lipinski Rule	Pfizer Rule	GSK
C1/Fucoxanthin	Antiobesity (0.908)	130	3	Rejected	Accepted	Rejected
C2/Lutein	Lipid metabolism regulator (0.805)	10	2	Rejected	Rejected	Rejected
C3/Astaxanthin	Lipid metabolism regulator (0.844)	4600	5	Rejected	Rejected	Rejected
C4/Canthaxanthin	Lipid metabolism regulator (0.821)	10,000	6	Rejected	Rejected	Rejected
C5/Zeaxanthin	Lipid metabolism regulator (0.936)	10	2	Rejected	Rejected	Rejected
C6/β-Cryptoxanthin	Lipid metabolism regulator (0.946)	10	2	Rejected	Rejected	Rejected
C7/β-Carotene	Lipid metabolism regulator (0.918)	1190	4	Rejected	Rejected	Rejected

* Way2Drug; ** Protox; *** ADMET.

**Table 4:** ΔG of molecular docking parameters of identified carotenoid from *E. acoroides*.

Receptors/Proteins (PDB ID)	Gibbs Free Energy (ΔG; kcal/mol)					
	C3	C4	C7	Control/Acarbose	Control/Orlistat	Control/S-Ibuprofen
iNOS (3E7G)	−9.3	−9.4	−9.7			−8.3
Lipase (1LPB)	−9.2	−9.7	−9.6		−7.1	
α-Glucosidase (3L4Y)	−8.0	−8.0	−8.0	−6.8		
α-Amylase (2QV4)	−10.7	−10.0	−9.7	−7.7		

C3, astaxanthin; C4, canthaxanthin; C7, β-carotene.

**Table 5:** Graphical depiction of the interaction between amino acids and the carotenoid identified from *E. acoroides* with specific receptor proteins.

Proteins	C3	C4
iNOS	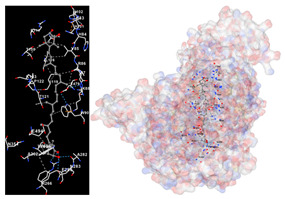	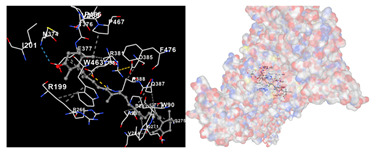
Lipase	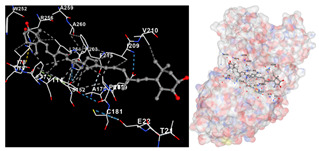	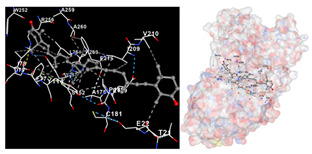
α-Glucosidase	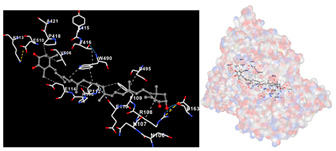	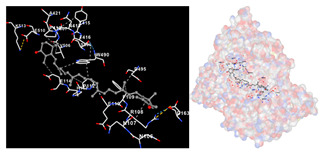
α-Amylase	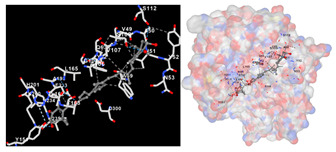	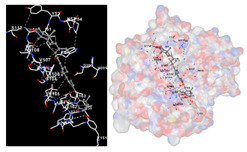

C3, astaxanthin; C4, canthaxanthin.

## Data Availability

The data presented in this study are available on request from the corresponding author.

## Supplementary Materials

The following supporting information can be downloaded at https://www.mdpi.com/article/10.3390/md22080365/s1, Table S1: The visual representation of the interaction between amino acids and the carotenoid identified from *E. acoroides* against specific receptor proteins; Table S2: The SMILES notation for each carotenoid of seagrass *E. acoroides* obtained from PubChem.
