# Supplementary material for: A Comparative Analysis on Impact of Extraction Methods on Carotenoids Composition, Antioxidants, Antidiabetes, and Antiobesity Properties in Seagrass Enhalus acoroides: In Silico and In Vitro Study
Source: Mar Drugs. 2024 Aug 12;22(8):365. doi: 10.3390/md22080365 (PMC11355445; doi:10.3390/md22080365)

| **Compound and Controls as Ligands** | iNOS (3E7G) |
| --- | --- |
| Astaxanthin (C3) | 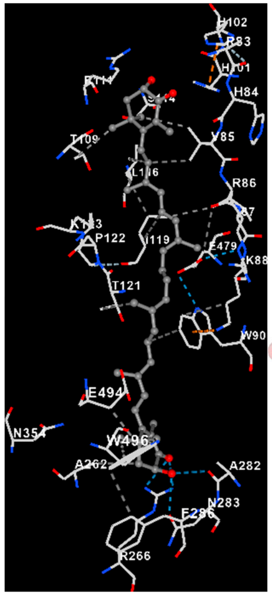 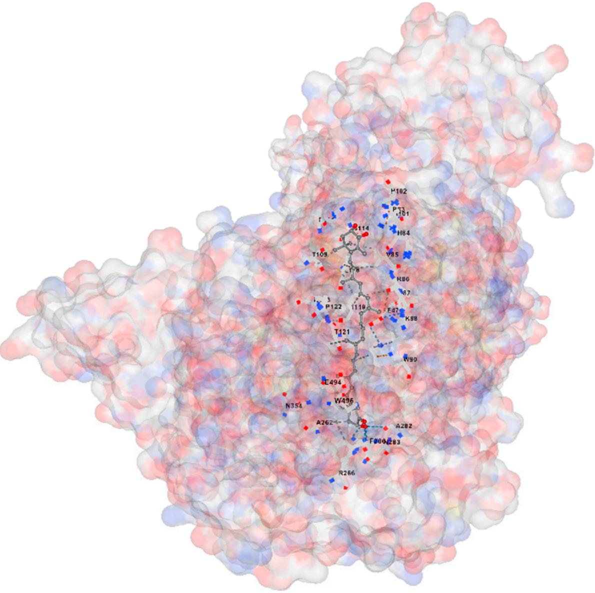 |

Canthaxanthin (C4)

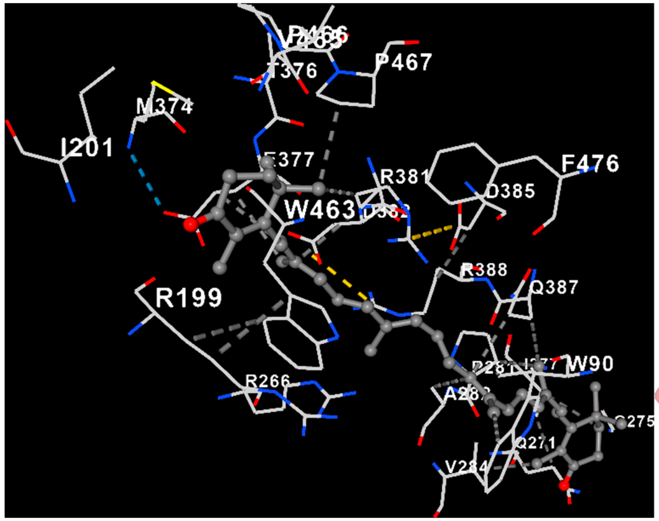


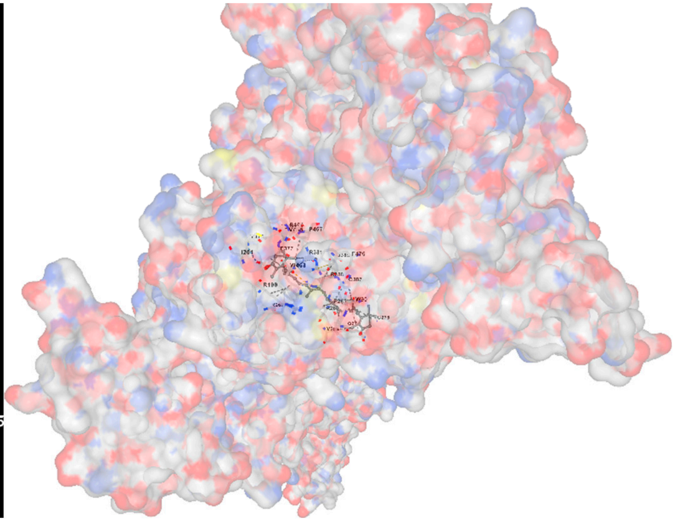

β-Carotene (C7)

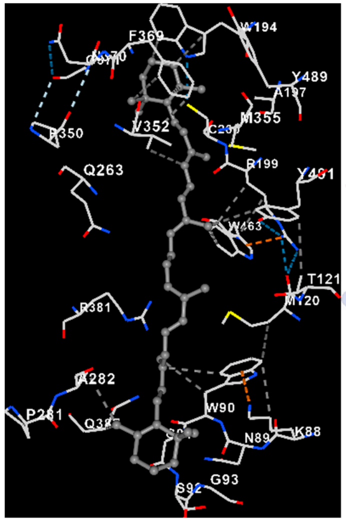


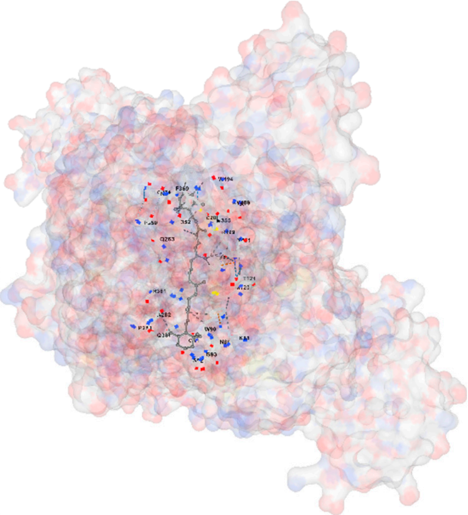

| | | |
|---|---|---|
| Acarbose | 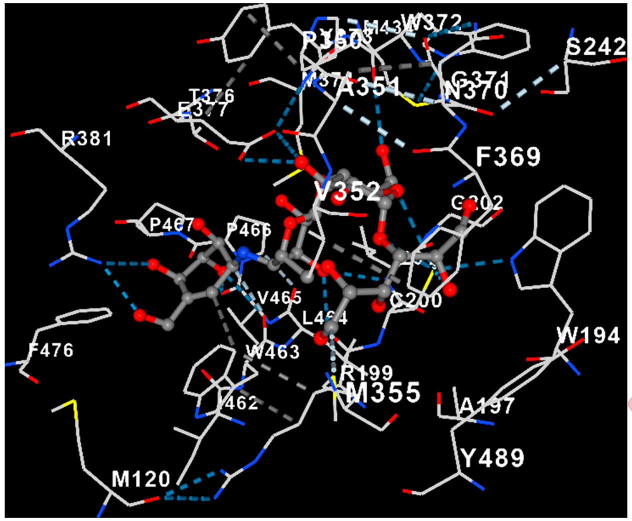 | 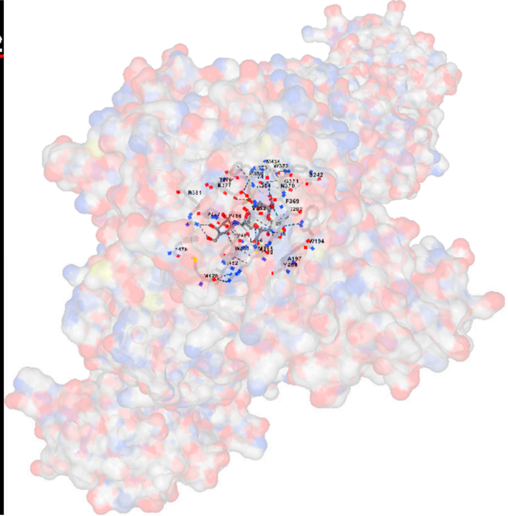 |
| Orlistat | 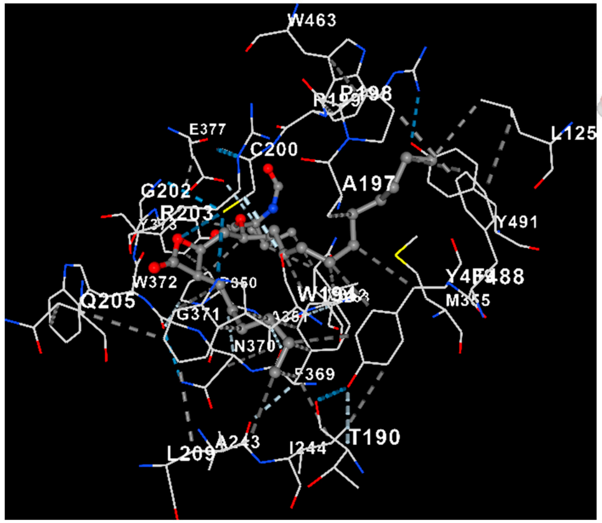 | 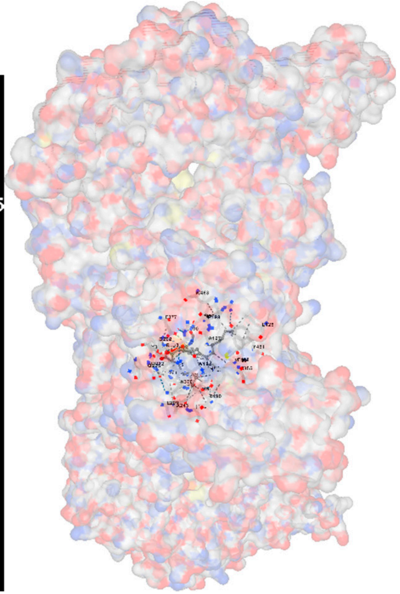 |

| | | |
|---|---|---|
| S-Ibuprofen | 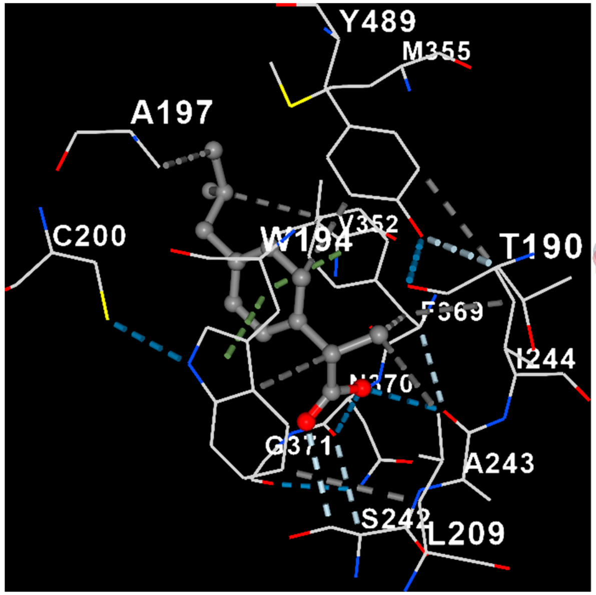 | 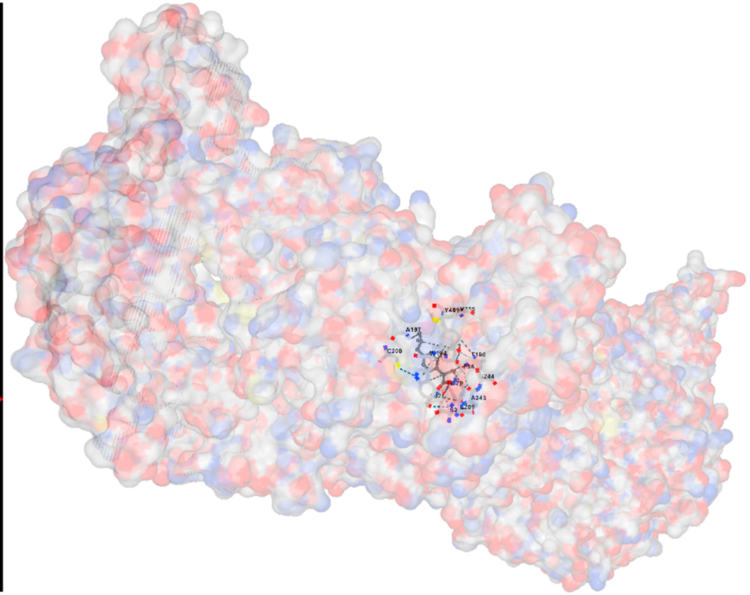 |


Y489
M355
A197
C200
W194
V352
T190
F369
I244
N370
G371
A243
S242
L209

| **Compound and Controls as Ligands** | Lipase (1LPB) |
| --- | --- |
| Astaxanthin (C3) | 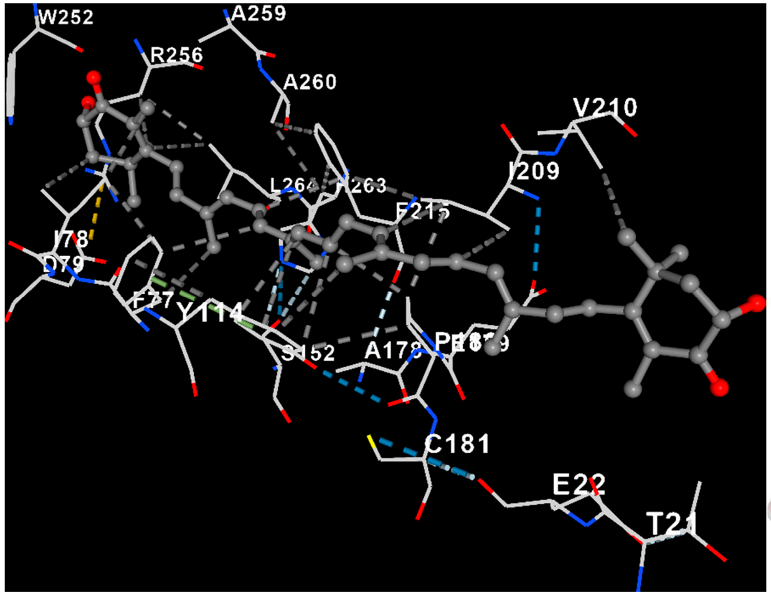  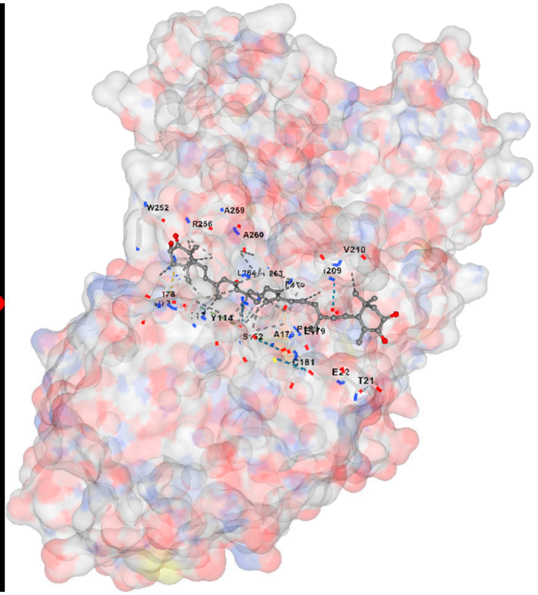 |

Canthaxanthin (C4)

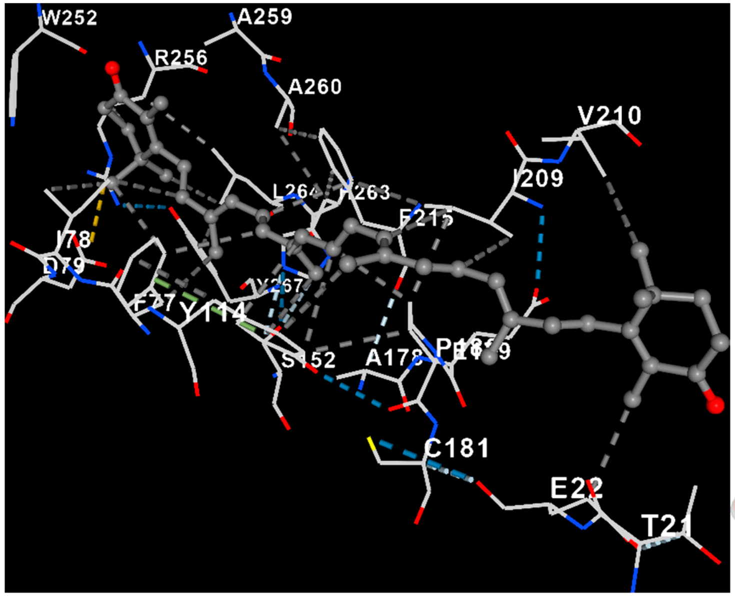


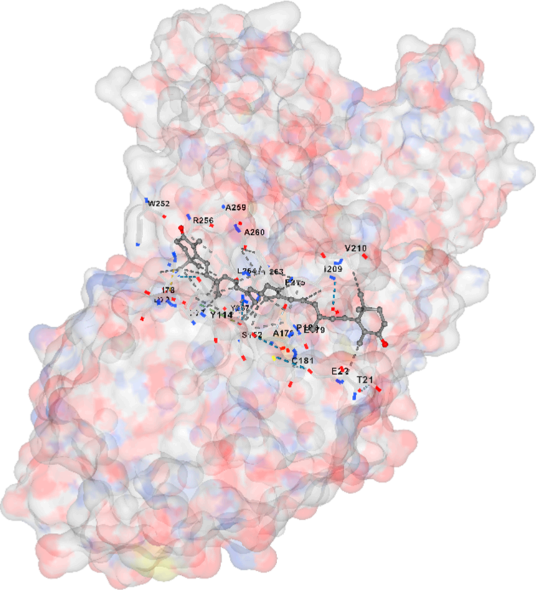

β-Carotene (C7)

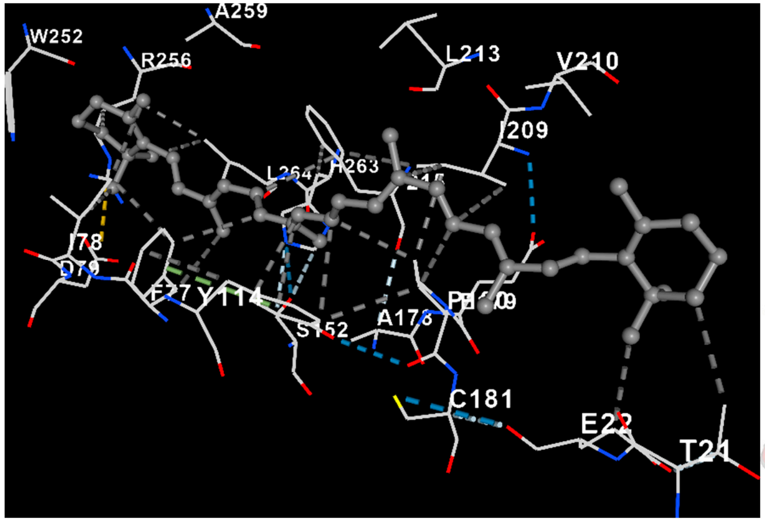


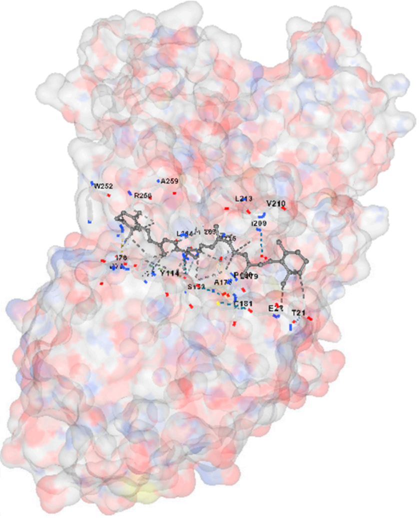

Acarbose

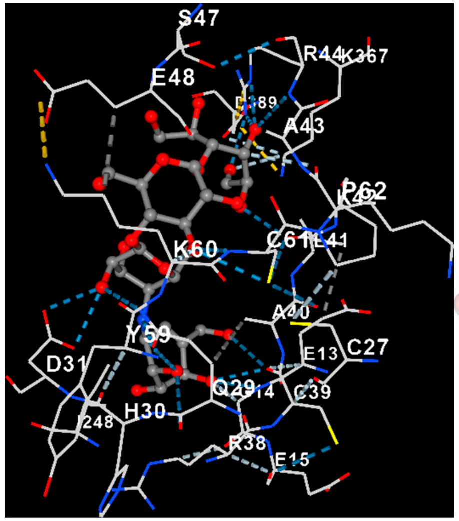


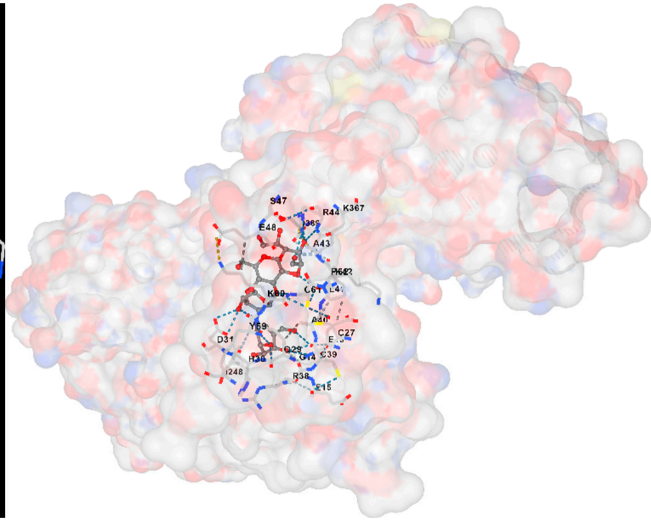

Orlistat

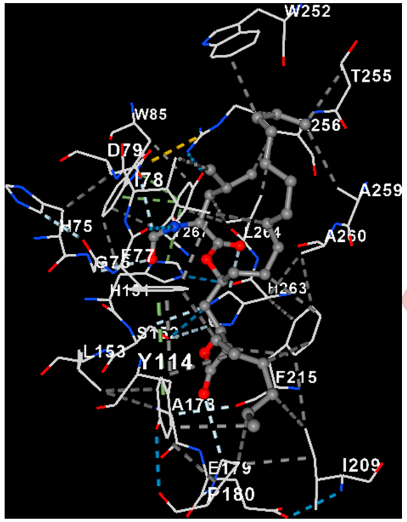


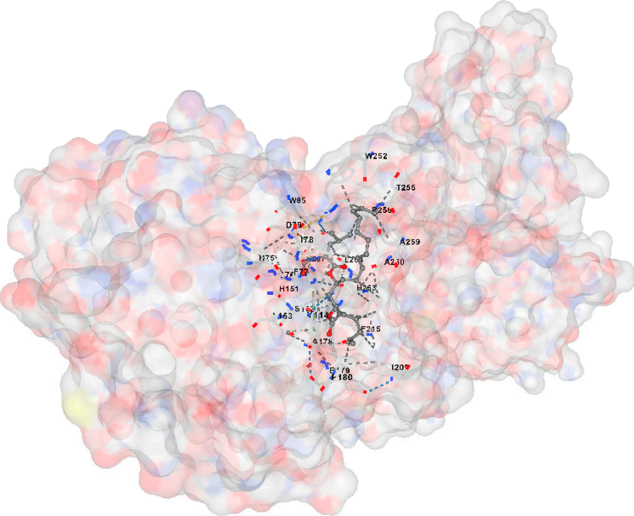

| | | |
|---|---|---|
| S-Ibuprofen | 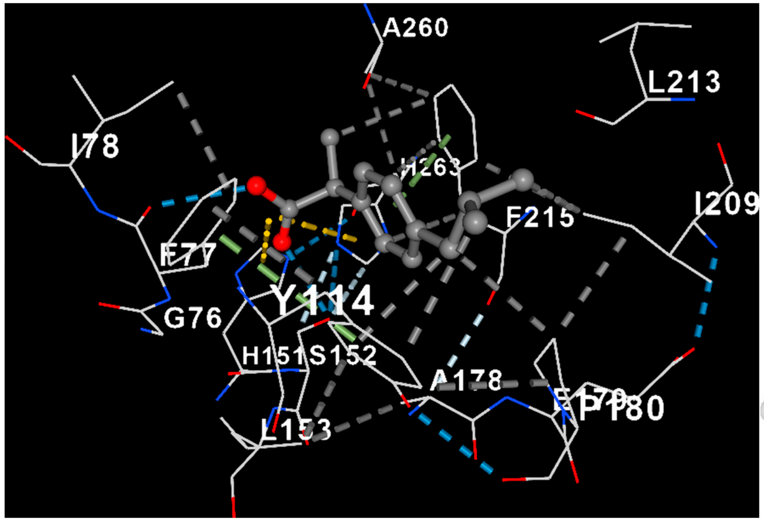 A260<br>L213<br>I78<br>H263<br>I209<br>F215<br>F77<br>Y114<br>G76<br>H151<br>S152<br>A178<br>P180<br>L153 | 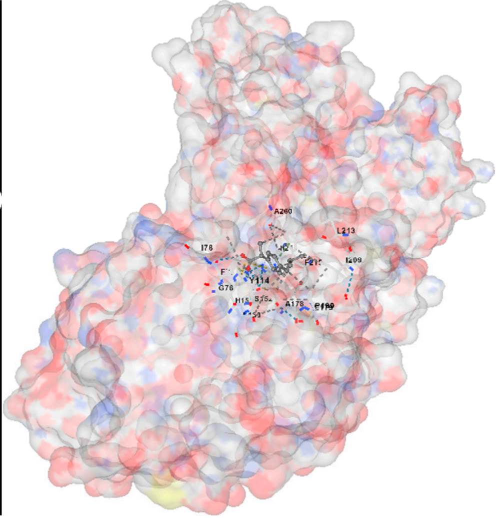 |

| **Compound and Controls as Ligands** | $\alpha$-Glucosidase (3L4Y) | |
|---|---|---|
| Astaxanthin (C3) | 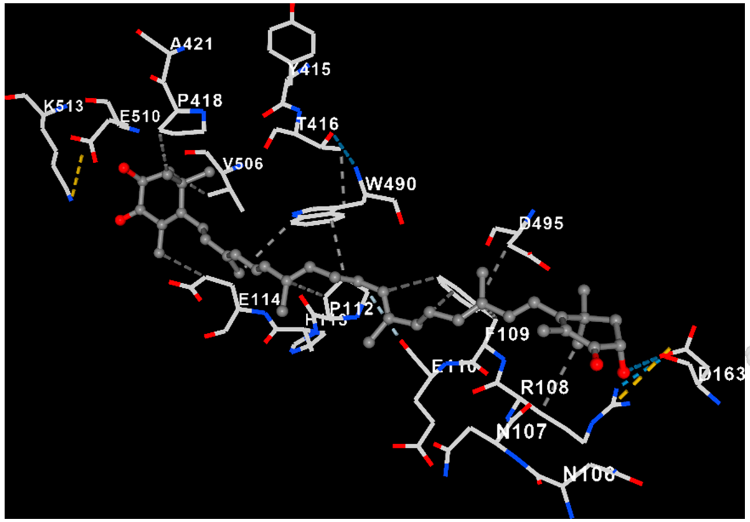 | 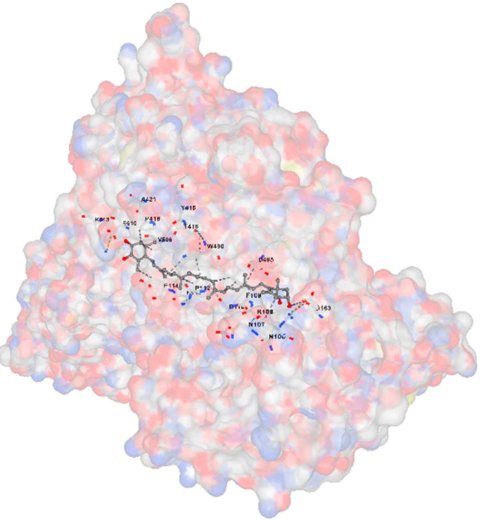 |
| Canthaxanthin (C4) | 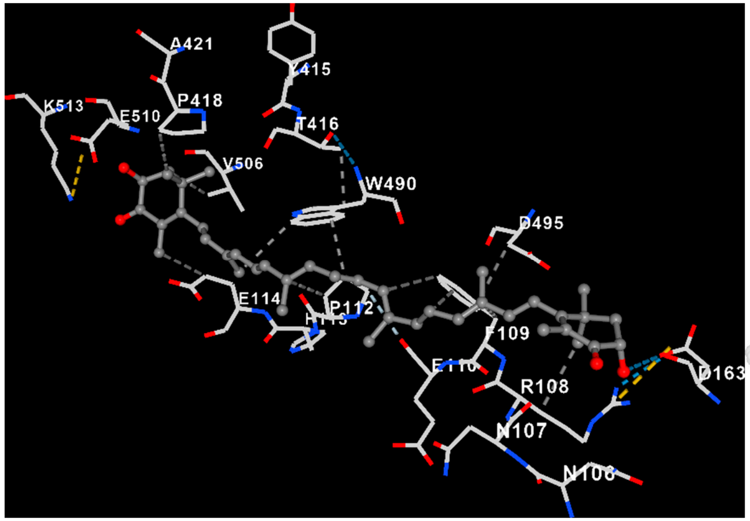 | 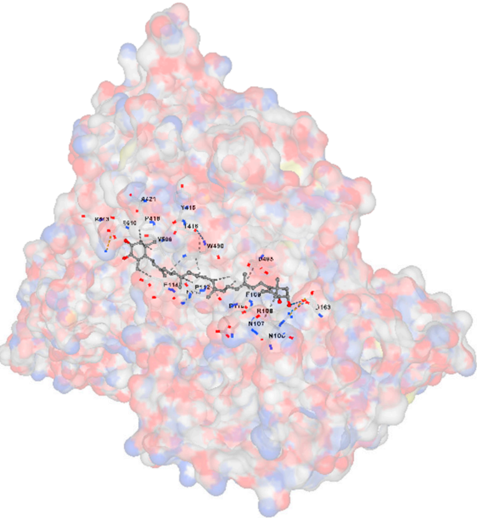 |

β-Carotene (C7)

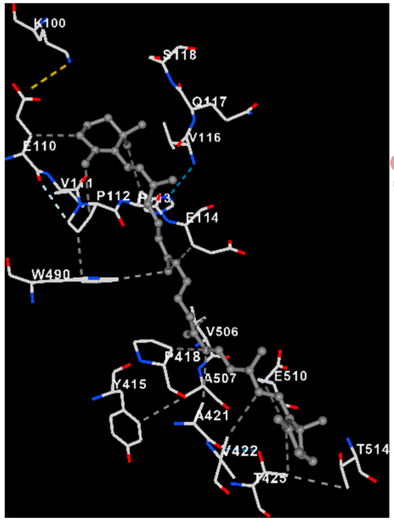


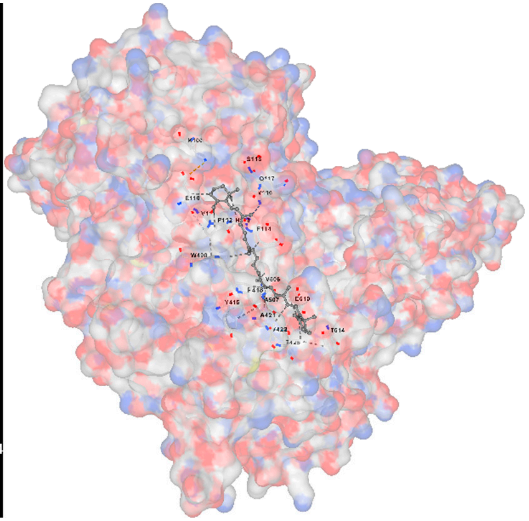

Acarbose

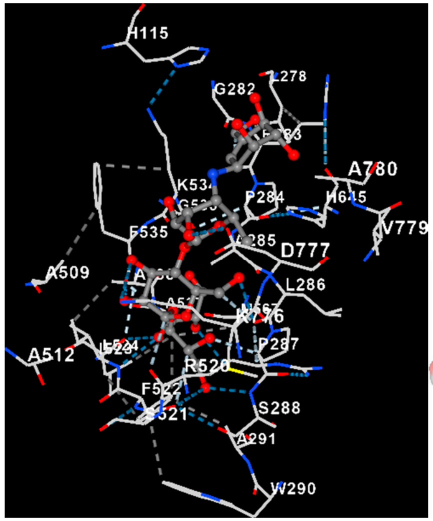


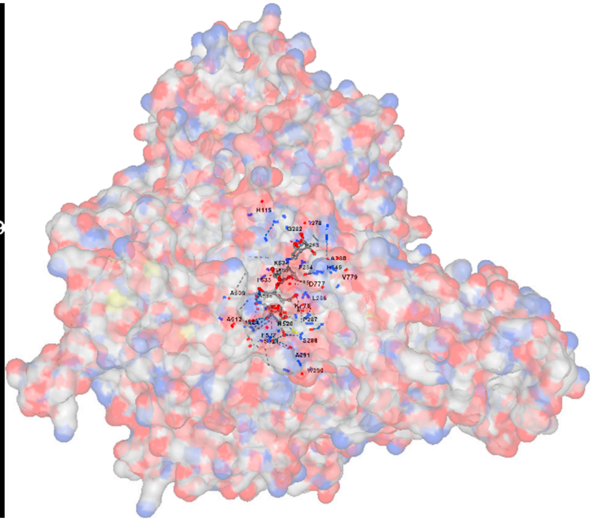

| | | |
|---|---|---|
| Orlistat | 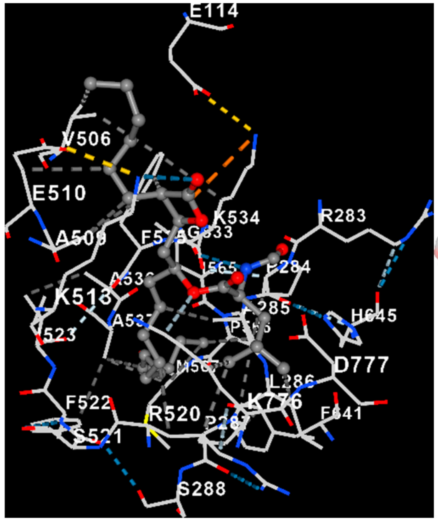 | 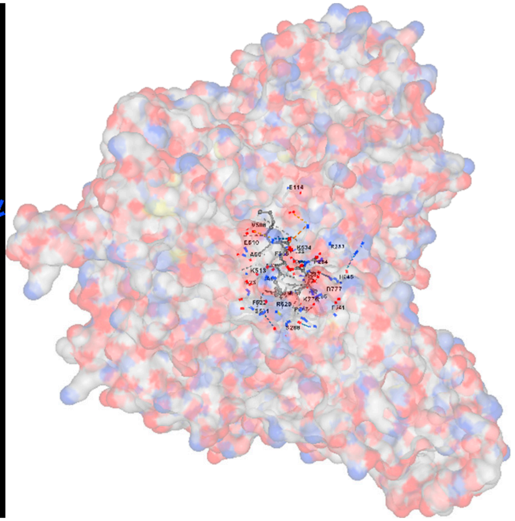 |
| S-Ibuprofen | 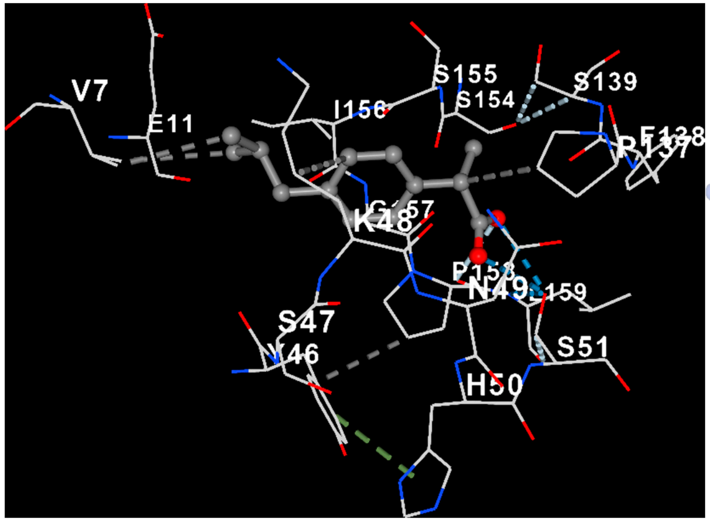 | 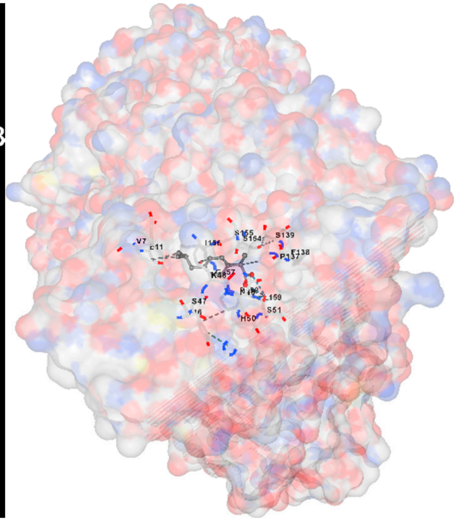 |

**Compound and Controls as Ligands** — $\alpha$-Amylase (2QV4)

Astaxanthin (C3)

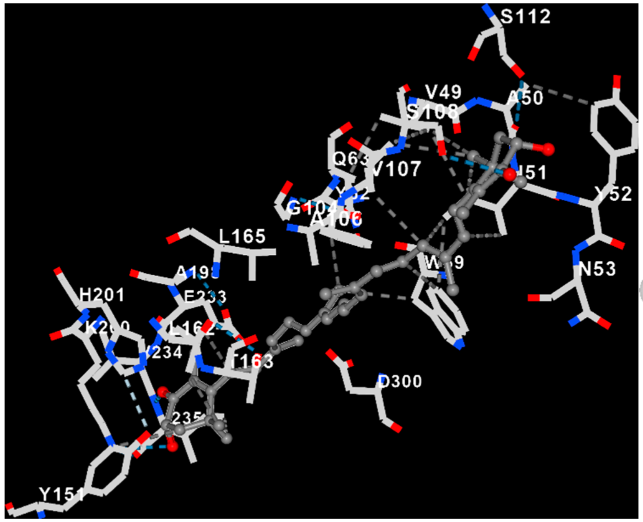


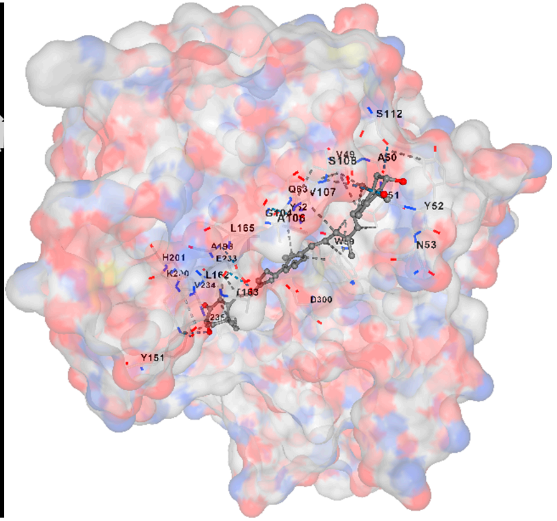

Canthaxanthin (C4)

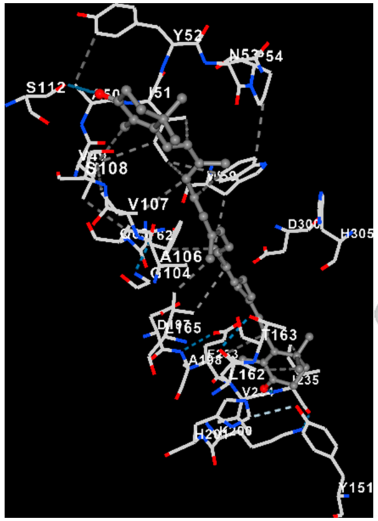


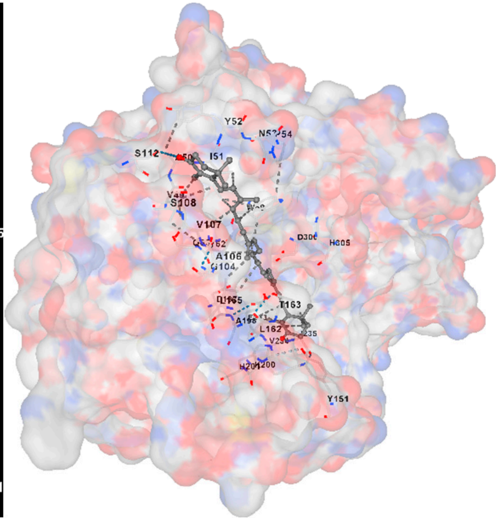

β-Carotene (C7)

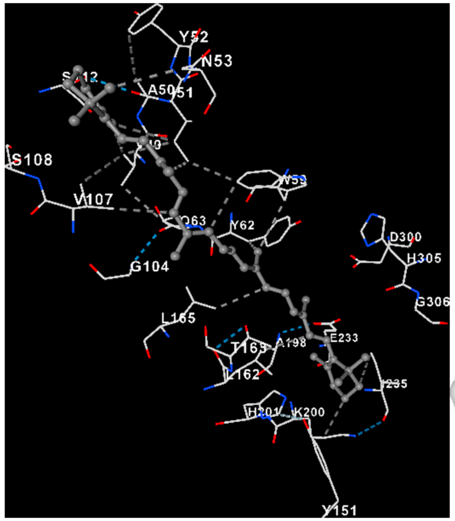


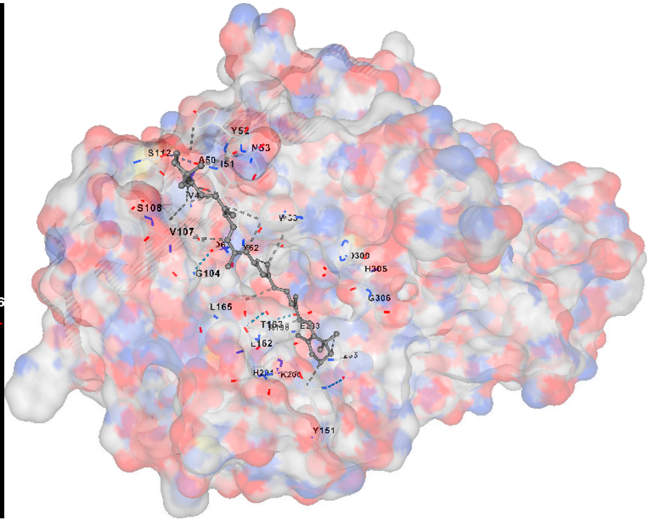

Acarbose

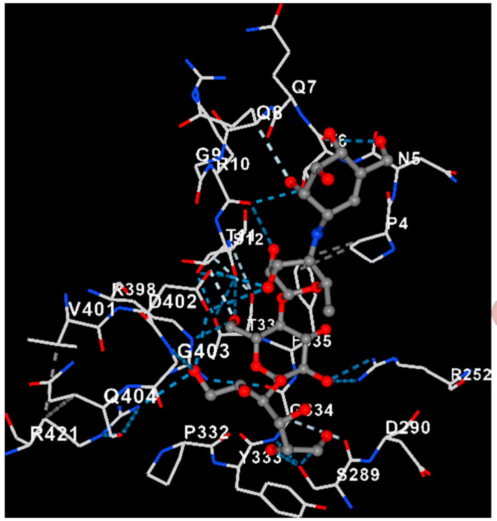


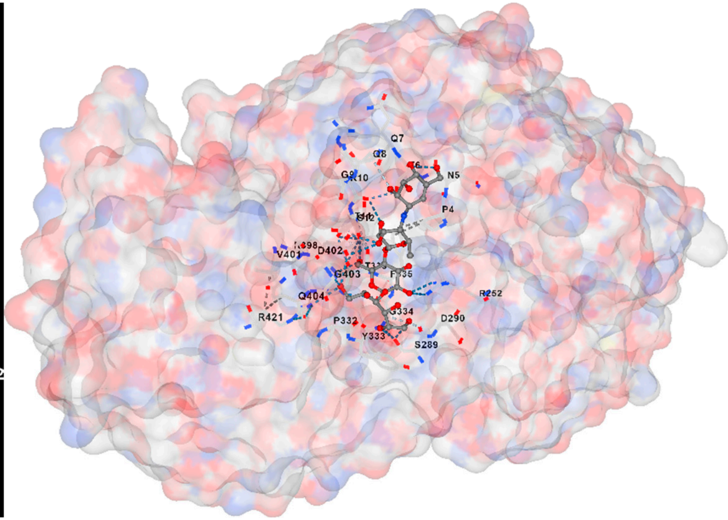

Orlistat

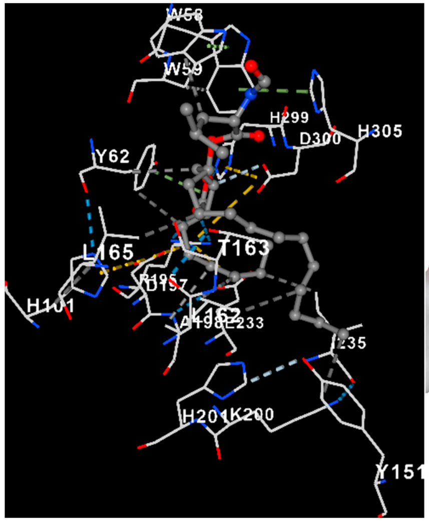


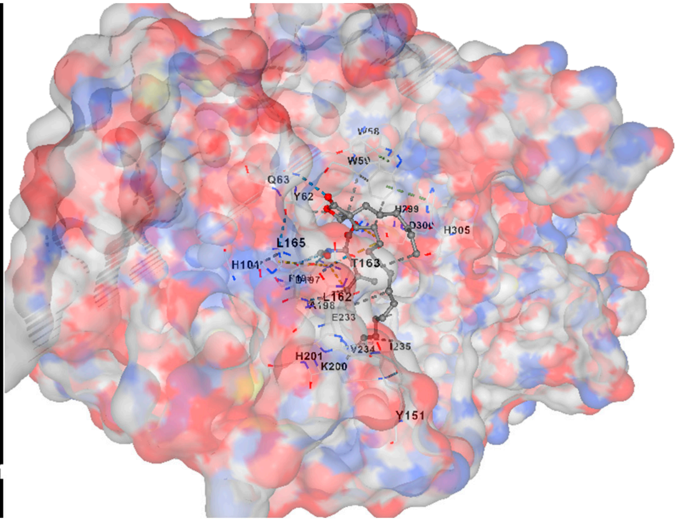


S-Ibuprofen

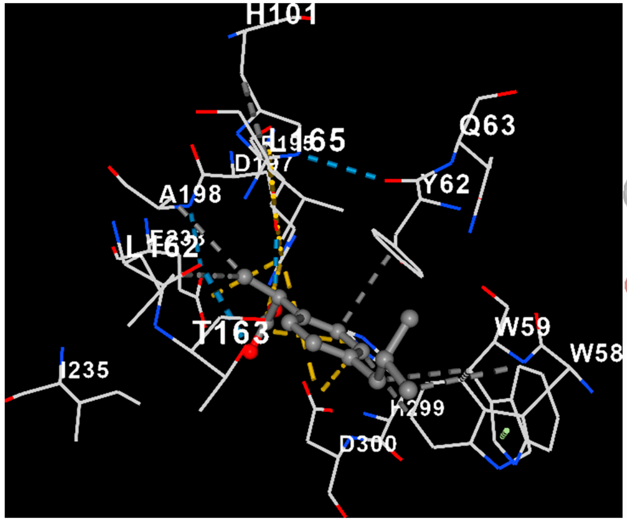


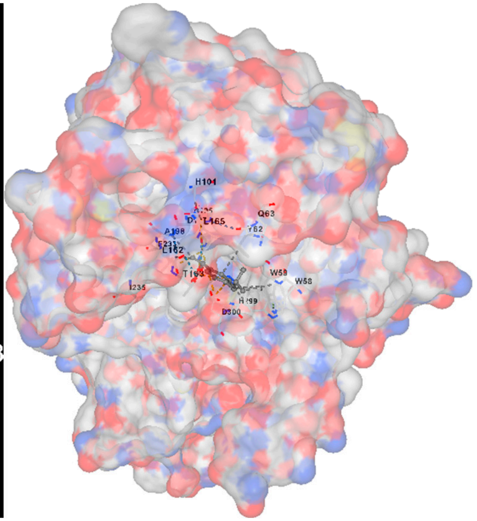

Supplement: Supplementary file 1 [file marinedrugs-22-00365-s001.zip › Table S1.pdf]
